# A multistate outbreak of Escherichia coli O157:H7 infections linked to alfalfa sprouts grown from contaminated seeds.

**DOI:** 10.3201/eid0706.010609

**Published:** 2001

**Authors:** T Breuer, D H Benkel, R L Shapiro, W N Hall, M M Winnett, M J Linn, J Neimann, T J Barrett, S Dietrich, F P Downes, D M Toney, J L Pearson, H Rolka, L Slutsker, P M Griffin, Investigation Team

**Affiliations:** Centers for Disease Control And Prevention, Atlanta, Georgia, USA. breuert@rki.de

## Abstract

A multistate outbreak of Escherichia coli O157:H7 infections occurred in the United States in June and July 1997. Two concurrent outbreaks were investigated through independent case-control studies in Michigan and Virginia and by subtyping isolates with pulsed-field gel electrophoresis (PFGE). Isolates from 85 persons were indistinguishable by PFGE. Alfalfa sprouts were the only exposure associated with E. coli O157:H7 infection in both Michigan and Virginia. Seeds used for sprouting were traced back to one common lot harvested in Idaho. New subtyping tools such as PFGE used in this investigation are essential to link isolated infections to a single outbreak.


### A Multistate Outbreak of *Escherichia coli* O157:H7 Infections Linked to Alfalfa Sprouts Grown from Contaminated Seeds

**Thomas Breuer,* Denise H. Benkel,*† Roger L. Shapiro,* William N. Hall,‡ Mary M. Winnett,§ Mary Jean Linn,† Jakob Neimann,* Timothy J. Barrett,* Stephen Dietrich,‡ Frances P. Downes,‡ Denise M. Toney,¶ James L. Pearson,¶ Henry Rolka,* Laurence Slutsker,* Patricia M. Griffin,* and the Investigation Team**[1]

*Centers for Disease Control and Prevention, Atlanta, Georgia, USA; †Virginia Department of Health, Richmond, Virginia, USA; ‡Michigan Department of Community Health, Lansing, Michigan, USA; §Medical College of Virginia, Richmond, Virginia, USA; and ¶Virginia Division of Consolidated Laboratory Services, Richmond, Virginia, USA

A multistate outbreak of *Escherichia coli* O157:H7 infections occurred in the United States in June and July 1997. Two concurrent outbreaks were investigated through independent case-control studies in Michigan and Virginia and by subtyping isolates with pulsed-field gel electrophoresis (PFGE). Isolates from 85 persons were indistinguishable by PFGE. Alfalfa sprouts were the only exposure associated with *E. coli* O157:H7 infection in both Michigan and Virginia. Seeds used for sprouting were traced back to one common lot harvested in Idaho. New subtyping tools such as PFGE used in this investigation are essential to link isolated infections to a single outbreak.

*Escherichia coli* O157:H7 was first recognized as a human pathogen in 1982 and has since emerged as a major cause of bloody and nonbloody diarrhea, causing thousands of infections with substantial illness and death each year in the United States (1). In addition, *E. coli* O157:H7 infection is the most common cause of hemolytic uremic syndrome, the leading cause of kidney failure among children in the United States.

Most foodborne outbreaks associated with *E. coli* O157:H7 have been traced to foods derived from cattle, especially ground beef and milk (1). In June and July 1997, the state health departments of Michigan and Virginia concurrently received an increased number of reports of *E. coli* O157:H7 infections compared with the numbers in similar periods the previous year. We describe the epidemiologic, environmental, and laboratory investigations that led to the identification of alfalfa sprouts grown from contaminated seeds as a new vehicle for *E. coli* O157:H7 infection in humans.

Address for correspondence: Thomas Breuer, Infectious Disease Epidemiology Section, Robert Koch-Institute, Stresemannstr. 90 – 102, 10963 Berlin, Germany; fax +49-30-4547-3533; e-mail: breuert@rki.de

### Methods

### Epidemiologic Investigation

Independent studies were done in Michigan and Virginia to investigate exposures associated with *E. coli* O157:H7 infection.

#### Michigan

Cases were identified through passive surveillance of isolates sent from clinical laboratories to the Michigan Department of Community Health (MDCH). During July 21-27, 1997, we conducted a case-control study in Michigan, using a questionnaire designed to test hypotheses developed during in-depth interviews with seven ill persons. Because alfalfa sprouts and salads were the most commonly mentioned items in the hypothesis-generating interviews, the

[1]Lisa Baker, Elizabeth Barrett, Faye Bates, Sydney Borkey, Happy Calloway, Lori Chabassol, Cynthia Chaos, Shirley Coleman, Patricia Culhane-Gizzi, Catherine Cummins, Bernadette Cunanan, Barbara Delaria, Kathryn Driscoll, Dena Ellison, Renee Field, Jose Gonzales, Crystal Groth, Robert Hackler, Pamela Hankison, Suzanne Jenkins, Jack Kress, Jan LaPierre, Henry Martin, Michael McMahan, Christopher Melchert, Christina Meyers, Grayson Miller, John Monroe, Jane Moore, Anne M. Patch, Edward Payne, Patricia Plander, Linda Rose, Betty S. Rouse, John Rullan, Sylvia Ryder, Susan Scott, Richard Snaman, Nancy Timmons, Janis Travers, Linda Vasquez, Susan Willis, Rosemary Wlaschin, Diane Woolard, Patricia Young, and Phyllis Young, Virginia Department of Health; Judith Carroll and Sally Henderson, Virginia Division of Consolidated Laboratory Services; Elizabeth Petrilack, Medical College of Virginia; Frederick Barham III, John Beers, Ryan Davis, Laurie Richards, and Doug Saunders, Virginia Department of Agriculture and Consumer Services; Stephanie Kordick, Joseph Reardon, and Joyce Reddington, North Carolina Department of Environment, Health, and Natural Resources; Jay White, United States Navy; Nancy Haas, Shawn McDermott, Kevin O'Brien, and Charlotte Wilkins, U.S. Food and Drug Administration; Andrew Al-Shab, Jaime Altamirano, Robert Barrie, K. Como-Sabetti, Mary Frances Dorman, Sonja Hrabowy, Greg Jennings, Norman Keon, Judith Kloss-Smith, Michael Kucab, Robert Martin, Linda Moshur, Kathy Parrott, Linda Reese, Billie Ritter, Barbara Robinson-Dunn, Majid Simonds, and Kenneth Wilcox, Michigan Department of Community Health; and J. Douglas Park, John Tilden, and Gerald Wojtala, Michigan Department of Agriculture.

questionnaire focused on these food items. Other food items known to be vehicles for *E. coli* O157:H7 infections were also included.

A case was defined as diarrhea, abdominal cramps, or both, in a resident of Michigan with onset of symptoms from June 15 to July 31, 1997, and a stool culture yielding *E. coli* O157:H7 with the outbreak strain pulsed-field gel electrophoresis (PFGE) pattern. Ill persons were interviewed by telephone about their illness and exposures during the 7 days before onset of symptoms. Two age group-, sex-, and neighborhood- (based on the same telephone prefix) matched controls were selected for each patient. Matching by age was based on six groups (≤2, 2 to <5, 5 to <12, 12 to <18, 18 to <60, and ≥60 years). Children <12 years of age were not matched by sex. Controls were identified by systematically adding to or subtracting from the case-patient's telephone number until a match was obtained. Controls were asked about food exposures during the 7 days before the day of interview. In addition, questions were asked about the consumption of selected food items such as alfalfa sprouts and salad during the time period including the 7 days before the onset of illness in the matched patient. The water quality records of a nearby lake where ill persons had swum were reviewed.

### Virginia

Cases were reported to the Virginia Department of Health by the local health districts, the state public health laboratory (Division of Consolidated Laboratory Services), and hospital and private laboratories. We initiated a case-control study on July 15, 1997, using a questionnaire regarding 31 specific food items and environmental exposures. The study design and questionnaire were not discussed with the Michigan investigators. Case-patients and controls were interviewed via telephone by trained interviewers who used a data collection instrument based on a Centers for Disease Control and Prevention (CDC) questionnaire used for a nationwide *E. coli* O157:H7 case-control study, with additional questions on items mentioned in interviews with ill persons. Controls were matched with case-patients by age, sex, and geographic location, with 1 to 3 controls per case. Case-patients <18 years old were matched within 3 years, those 18 to 34 years old within 5 years, and those >34 years old within 10 years. To locate geographically matched controls, investigators dialed the case-patients' area code and the first five digits of his or her telephone number and then completed the call by using a list of randomly generated numbers for the last two digits.

A confirmed case was defined as diarrheal illness (three or more loose stools in a 24-hour period) occurring in a Virginia resident (or nonresident if the person was seen in a Virginia health-care facility for treatment) with onset of symptoms from June 1 to September 5, 1997, and a stool culture that yielded *E. coli* O157:H7 with the outbreak strain PFGE pattern. Potential controls were excluded if they reported having had diarrhea in the 2 months before the interview or if they were not living in their current residence during the week before the matching patient's onset of illness. Controls were asked about the same time period as the matched patient. If case-patients or controls could not remember eating an item during that week, they were asked whether that item would have been eaten in a typical week in the month of the patient's illness onset.

### Laboratory Methods

PFGE subtyping of *E. coli* O157:H7 isolates was performed by the MDCH laboratory and the Virginia State laboratory by using the restriction enzyme *Xba*I (Boehringer Mannheim, Indianapolis, IN) as described (2). A subset of isolates (12 from Michigan and 24 from Virginia) were sent to CDC for simultaneous PFGE subtyping, phage typing, and antimicrobial susceptibility testing. PFGE subtyping of selected isolates was also performed by using the restriction enzymes *Bln*I (Boehringer) and *Spe*I (Boehringer).

In addition, we asked all state and territorial epidemiologists and public health laboratory directors to send to CDC *E. coli* O157:H7 isolates from any patients who had eaten alfalfa sprouts in the week before illness.

Isolates with PFGE patterns indistinguishable from the predominant pattern of isolates in Michigan were considered the outbreak strain. The outbreak PFGE pattern was compared with PFGE patterns in the CDC database by Molecular Analyst Fingerprinting Plus Software (Bio-Rad Laboratories, Hercules, CA). Phage typing was done by the extended phage typing scheme of Khakhria et al. (3). Isolates were tested by the disk-diffusion technique for susceptibility to the following antimicrobial agents: amoxicillin/clavulanic acid, ampicillin, ceftriaxone, chloramphenicol, ciprofloxacin, gentamicin, kanamycin, nalidixic acid, streptomycin, sulfisoxazole, tetracycline, and trimethoprim-sulfamethoxazole (4).

Implicated alfalfa seed and sprouts grown from that seed were cultured for *E. coli* O157:H7 in two enrichment broths (mTSB and TSBcv) (5,6). The broths were streaked to Sorbitol MacConkey Agar (CT-SMAC) containing 0.05 mg/L cefixime and 2.5 mg/L potassium tellurite) and further examined by immunomagnetic separation (anti-*E. coli* O157 Dynabeads; Dynal, Inc., Lake Success, NY.) (7). The concentrated samples were then plated to selective media (CT-SMAC). Sweeps of growth on all plates were tested by polymerase chain reaction for stx1, stx2, and uidA gene sequences (8,9).

### Trace-Back Methods

State health departments, in conjunction with the Department of Agriculture in Michigan, the local Food and Drug Administration in Virginia, and CDC investigators, conducted interviews to determine the source(s) of sprouts. Ill persons identified retail outlets (restaurants, markets) and dates of purchase. Retail outlets identified shippers and growers of alfalfa seed. The origin of implicated seed was determined by reviewing seed supplier invoices and delivery records. A successful trace-back from a patient who had eaten sprouts was defined as one in which the grower and seed supplier(s) could be identified. After the alfalfa sprouts were traced back to one specific lot of seeds, we investigated the seed processing company and the fields from which they were harvested.

### Statistical Methods

Maximum likelihood estimates of matched odds ratios (MOR) with exact 95% confidence intervals (CI) were used as measures of association (SAS statistical software version 6.12, SAS Institute Inc., Cary, NC, USA).

### Results

### Epidemiologic Investigation

#### Michigan

From June 1 to July 31, 64 persons with *E. coli* O157:H7 infection from Michigan were reported to MDCH, a twofold increase from the 31 infections reported during the same 2 months in 1996. Thirty-eight patients had illness that met the case definition (Figure). Of these, 26 (68%) were female, and the median age was 31 years (Table). Ninety-five percent reported bloody diarrhea, 47% were hospitalized, and 11% had hemolytic uremic syndrome; none died. Sixty-six percent of patients reported that they received antibiotics; 74% reported the use of antimotility agents.

Twenty-seven case-control sets were interviewed; the remaining patients either could not be reached or were identified after the study ended. The only food item positively and significantly associated with illness was alfalfa sprouts. Fifteen (56%) of 27 ill persons reported eating alfalfa sprouts in the 7 days before onset of illness, but only 3 (6%) of 53 controls had eaten them in the 7 days before the interview (MOR 27; 95% CI 5-558). When controls were asked about alfalfa sprout consumption for the same 7-day interval as ill persons, a similar association was observed (4 [8%] of 53 controls; MOR 25; 95% CI 4-528). No other food item–or swimming—was positively associated with illness.

#### Virginia

In June 1997, 32 persons with *E. coli* O157:H7 infection were reported, compared with 11 cases in the same time period in 1996. Seventy-four persons with *E. coli* O157:H7 infection with dates of onset from June 1 to September 5, 1997, were reported. Forty-four (59%) had illnesses that met the case definition; the exact onset dates were reported for 42 (Figure). Demographic and clinical characteristics of patients were similar to those of Michigan patients (Table).

Twenty case-control sets could be contacted for the case-control study. As in Michigan, eating alfalfa sprouts was the only exposure positively and significantly associated with illness. Thirteen (68%) of 19 case-patients but only 6 (13%) of 45 controls reported eating alfalfa sprouts during the week before illness onset in the case or in a typical week in the month of the patient's illness onset (MOR 25; 95% CI 4-537). No other food item was significantly associated with illness.

### Laboratory Results

In the subset of 34 *E. coli* O157:H7 isolates from case-patients in Michigan and Virginia submitted to CDC, all isolates had indistinguishable PFGE patterns with restriction enzyme *Xba*I and *Bln*I. Three isolates were also tested by

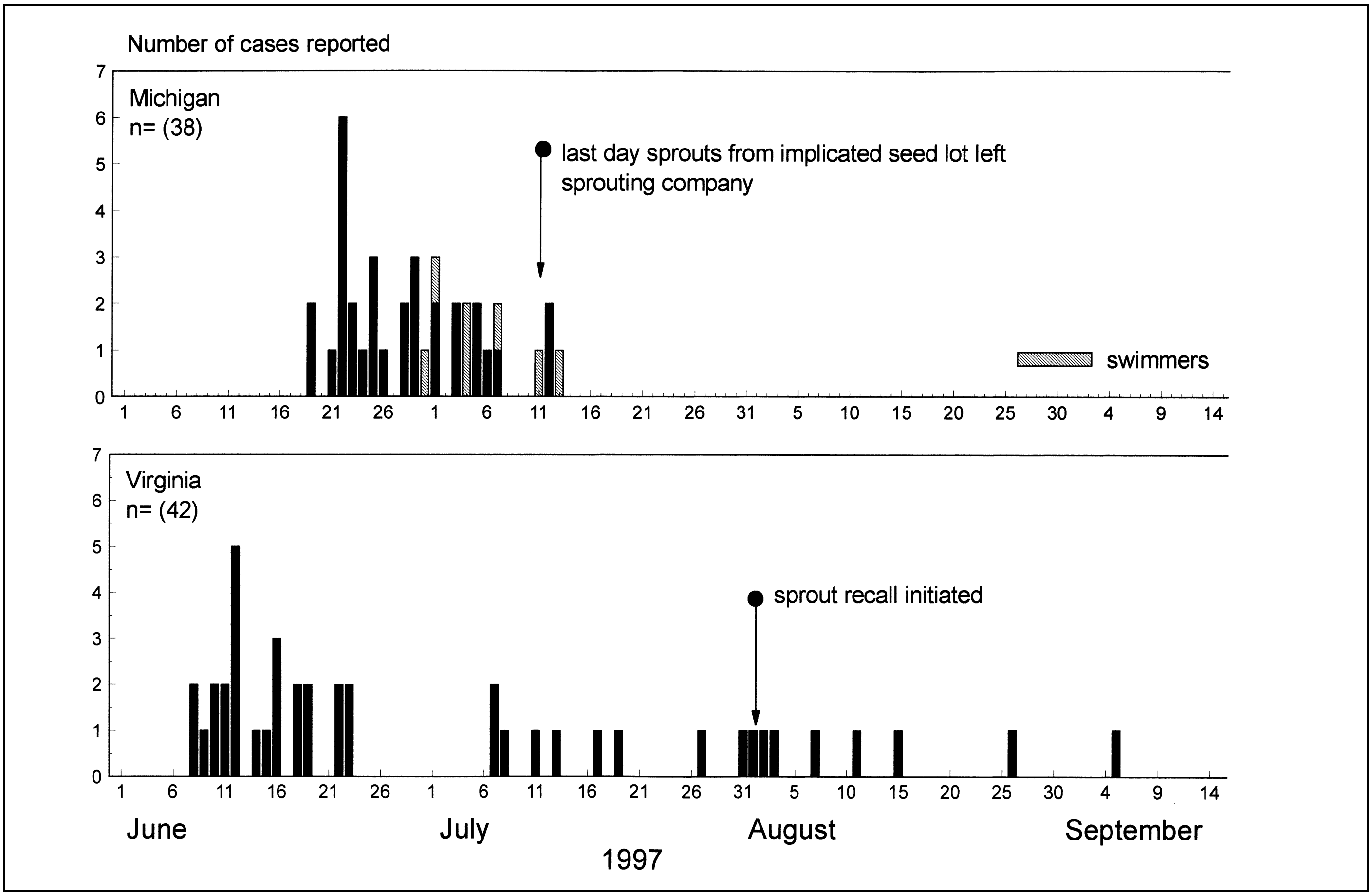

Figure. Date of onset of illness for persons with *Escherichia coli* O157:H7 infection and the outbreak pulsed-field gel electrophoresis pattern, Michigan and Virginia, June to September 1997.

Table. Demographic and clinical characteristics of persons with *Escherichia coli* O157:H7 infections and the outbreak pulsed-field gel electrophoresis patterns, Michigan and Virginia, 1997

| Characteristic | Michigan (n=38) | Virginia (n=44)[a] |
|---|---|---|
| Median age, years (range) | 31 (2-76) | 33 (1-71) |
| Female, no. (%) | 26 (68) | 26 (59) |
| Signs and symptoms, no. (%) | | |
| Diarrhea | 38 (100) | 44 (100) |
| Abdominal cramps | 37 (97) | 40 (100) |
| Bloody stool | 36 (95) | 38 (95) |
| Vomiting | 20 (53) | 26 (62) |
| Subjective fever | 15 (39) | 15 (43) |
| Hospitalized | 18 (47) | 18 (49) |
| HUS[b] | 4 (11) | 0 (0) |
| Headache | ND | 18 (47) |
| Muscle aches | ND | 15 (44) |
| Median days of diarrhea, no. (range) | 6 (3-41) | ND |

[a]Denominator ranged from 34 to 44 because of missing information.
[b]HUS = hemolytic uremic syndrome; ND = not determined.

*Spe*I and had indistinguishable patterns. All isolates were phage type 32. Thirty-three isolates were sensitive to all antimicrobial agents tested; one isolate from Virginia was resistant to ampicillin.

Sixty-seven isolates collected during the outbreak period from U.S. states other than Michigan and Virginia were tested at CDC by PFGE by using *Xba*I, and *Bln*I, and *Spe*I restriction enzymes. Six (9%) had PFGE patterns indistinguishable from the outbreak strain PFGE pattern by all three enzymes and were phage type 32, bringing the total number of cases to 85. These isolates came from Ohio and North Carolina, which are directly adjacent to the outbreak states.

We obtained a 50-pound bag of seeds used to grow the implicated alfalfa sprouts. A 500-g sample of seeds was cultured directly, and the same amount of seeds was sprouted; neither yielded *E. coli* O157:H7.

### Trace-Back

Trace-back to the sprouting facility was successful in 29 of 31 instances in which ill persons reported eating alfalfa sprouts. Of 16 successful trace-backs in Michigan, 15 led to one sprouting facility, facility A, in Michigan; one patient could have eaten sprouts from either facility A or facility B in Michigan. All 13 successful trace-backs in Virginia were traced to one sprouting facility in Virginia. During the outbreak period, the Virginia company used only one seed lot. That same seed lot was one of only two lots continuously sprouted by facility A in Michigan from mid-May to the first week of July. Facility B in Michigan sprouted a small number of seeds from this lot on only 2 days; the sprouts from these seeds represented only a fraction of each day's production. The implicated seed lot was not distributed to any other sprouting company in or outside the United States. That seed lot was 17,000 lbs, of which 6,000 lbs still existed and were immediately removed from distribution.

The implicated seed lot was a blend of five lots from four farms, harvested from 1984 to 1996. The seed processor and the farmers were all located in Idaho. Inspection of the alfalfa fields revealed three possible sources of contamination: cattle manure, water, and deer feces. Manure is not normally applied in alfalfa fields in Idaho; however, cattle feedlots are common in the area. The alfalfa fields of one of the farmers were adjacent to a feedlot. Manure may have leaked or been illegally dumped next to feedlots. In addition, run-off water from neighboring fields, which is collected in furrows and sometimes used to irrigate alfalfa fields, could carry manure to the fields. Three of the four farmers reported at least occasionally seeing deer in their fields. In fact, one had fields next to a wildlife refuge and reported that deer were in the fields every day. Contaminated alfalfa plants, cattle manure, or deer feces could be picked up by the thresher during harvesting and contaminate the seeds. No evidence was found for bacterial contamination at the seed processor.

### Other Sources of Illness

We interviewed 11 patients in Michigan who met the case definition but were not included in the case-control study because they either could not be contacted during the study or had not been identified when the case control-study was conducted. The median age of these patients was 12 years, compared with 31 years for patients in the case-control study. Their onsets of illness were from June 30 to July 13. Five of these patients, all children, had definitely not eaten alfalfa sprouts but had swum in the same manmade lake during the July Fourth holiday weekend or the previous weekend.

We re-interviewed the eight patients who were enrolled in the case-control study and who reported swimming in the 7 days before illness. We identified two children (ages 4 and 5) who had not eaten alfalfa sprouts in the week before illness but who had been swimming at the same manmade lake during the same time period. None of the other patients had visited the lake. The number of visitors per day was not higher on these weekends than the average number for weekend days in June and July. Water samples from three locations in the beach area of the lake contained $\leq$ 10 *E. coli* bacteria/100 mL on June 25 and July 7.

Demographic characteristics of patients in Virginia (n=21) who were not included in the case-control study did not differ from those of patients enrolled in the study.

### Follow-Up of Patients Outside the Outbreak States

We contacted all six ill persons from North Carolina and Ohio who were infected with *E. coli* O157:H7 of the outbreak strain PFGE pattern. Two of them had traveled to an outbreak state during the outbreak period but could not remember eating alfalfa sprouts. Three remembered eating alfalfa sprouts in the week before illness but did not recall traveling to an adjacent outbreak state during that time.

### Discussion

This multistate outbreak of *E. coli* O157:H7 infections is the first outbreak linked to consumption of alfalfa sprouts. It is also the first outbreak in which subtyping by PFGE was used to determine the magnitude of the outbreak on a national scale. Many lines of evidence indicate that the vehicle was alfalfa sprouts grown from contaminated seed. First, two independently designed and conducted case-control studies of concurrent outbreaks in Michigan and Virginia found that eating alfalfa sprouts was the only exposure positively associated with illness. Second, *E. coli* O157:H7 isolates from Michigan and Virginia patients epidemiologically linked to the outbreak had an indistinguishable PFGE pattern, the same phage type, and the same antibiogram, strongly suggesting a common source. The identical PFGE pattern had been identified only once before in one isolate in CDC's database of PFGE patterns. Third, trace-back investigations implicated independently operating sprouting facilities in Michigan and Virginia. The only common link between these sprouting facilities was the use of the same seed lot, grown and shipped from Idaho, indicating that the seed was contaminated before it was shipped to the sprouting facilities. Fourth, the outbreak ended after alfalfa sprouts from the implicated seed lot were no longer sold. Fifth, a national sample of other *E. coli* O157:H7 isolates collected during the outbreak period did not contain the PFGE outbreak pattern, except for a few isolates from ill persons in states adjacent to the outbreak states, who could have eaten the implicated sprouts. The lack of a nationwide distribution of cases is consistent with the fact that the seeds and the sprouts grown from them were distributed in only two states.

This outbreak also highlights the use of PFGE subtyping as a tool for differentiating between an increase of sporadic unlinked infections and a cluster of infections from a common source. After an increased number of reports of *E. coli* O157:H7 infections was noticed, rapid PFGE subtyping of the initial isolates within 2 days was the starting point of this investigation.

The removal of 6,000 pounds of remaining seeds from the marketplace likely prevented more illnesses, although cultures of implicated seeds and sprouts grown from them did not yield *E. coli* O157:H7. Contamination was probably not uniform in the lot of seed from which the implicated sprouts were grown, and since only a single bag, representing <0.003% of that seed lot, was cultured, recovery of *E. coli* O157:H7 may have been unlikely. This outbreak investigation illustrates the recommendation that public health officials should not require confirmation of microbial contamination of a product before taking action when sufficient epidemiologic evidence is available.

In recent years, produce items such as lettuce, apple cider, and unpasteurized apple juice have been implicated in outbreaks of *E. coli* O157:H7 infections (10). Detection of non-meat-related outbreaks is most likely explained by heightened awareness of *E. coli* O157:H7 infection and improved detection methods such as PFGE subtyping, which enable recognition of smaller clusters and widely dispersed outbreaks. The expanded range of food vehicles also highlights the need for changes in educational messages and for improving awareness among physicians and the general public that *E. coli* O157:H7 infections can be acquired from many sources other than ground beef.

In recent years, consumption of raw alfalfa sprouts has also been associated with outbreaks due to various serotypes of *Salmonella* (11,12). Salmonellae can survive for months under the dry conditions used for alfalfa seed storage (13), and *E. coli* O157:H7 likely follows a similar survival pattern. Furthermore, *E. coli* O157:H7 proliferates $10^3$- to $10^5$-fold during sprout germination. Viable *E. coli* O157:H7 can exist not only on the outer surface but also inside the sprout vessels (14). If this finding is confirmed by other researchers, it is even more important to identify sources of contamination and institute specific prevention measures.

The U.S. sprouting industry produces several hundred thousand tons of sprouts of different varieties each year. No methods to reduce or eliminate contamination of seeds in the field, to effectively decontaminate alfalfa seeds before sprouting, or to clean the sprouts themselves are in place. The sprout industry is working with the National Center for Food Safety and Technology to study sprout safety. The most promising method is chemical treatment with calcium hypochlorite, a method already in use in California on an emergency basis, as approved by the state's environmental protection agency (15). Irradiation, in which a measured dose of ionizing radiation is applied, appears to work well in decontaminating sprout seeds; U.S. Food and Drug Administration approval is pending (15). Until the safety of alfalfa sprouts can be assured, we recommend that persons at increased risk for *E. coli* O157:H7 infections, such as children <5 years of age, the elderly, and patients with compromised immune systems, should not eat alfalfa sprouts (16,17).

This outbreak shows how foodborne outbreaks can extend in a community. The identification of an identical PFGE pattern in a second cluster of patients at the end of the outbreak suggests that a lake was contaminated by feces from a patient with illness from sprouts. Such contamination is possible because *E. coli* O157:H7 can survive for weeks in lake water (18) and has a very low infectious dose. Children could have acquired illness by swallowing a small amount of water while swimming (19,20). The finding of a probable waterborne outbreak also underscores the fact that new subtyping methods such as PFGE are tools to improve investigations but cannot substitute for a thorough epidemiologic work-up. Epidemiologic investigation combined with PFGE made the link to the source of the outbreak and to a specific lot of alfalfa seeds. Additional epidemiologic inquiry established lake water as the most likely mode of transmission for children who had not eaten sprouts.

Because food is increasingly centrally produced and widely distributed (21), the public health system will likely face more widely dispersed outbreaks such as this one. To meet the challenge, subtyping tools such as those used in this investigation, as well as timely and centralized disease reporting and outbreak investigation by trained public health personnel, the application of appropriate microbiologic tests in suspected cases, and increased awareness of foodborne illness will be essential.

### Acknowledgment

We thank Lynne McIntyre and Aparajita Singh-Breuer for editorial assistance in the preparation of this manuscript.

Dr. Breuer, a physician and epidemiologist, is head of the infectious disease epidemiology unit at the Robert Koch Institute in Berlin, Germany. He has a strong interest in infectious disease outbreak investigation. His responsibilities include national surveillance, outbreak investigations, and training.

### References

1. Mead P, Griffin PM. *Escherichia coli* O157:H7. Lancet 1998;352:1207-12.
2. Barrett TJ, Lior H, Green JH, Khakhria R, Wells JG, Bell BP, et al. Laboratory investigation of a multistate food-borne outbreak of *Escherichia coli* O157:H7 by using pulsed-field gel electrophoresis and phage typing. J Clin Microbiol 1994; 32:3013-7.
3. Khakhria R, Duck D, Lior H. Extended phage-typing scheme for *Escherichia coli* O157:H7. Epidemiol Infect 1990;105:511-20.
4. National Committee for Clinical Laboratory Standards. Performance Standards for Antimicrobial Susceptibility Testing; Eighth Informational Supplement. Wayne (PA): The Committee; 1998. NCCLS document M100-S8 [ISBN 1-56238-337-X]. NCCLS, 19087-1898.
5. Dohle MP, Schoeni JL. Isolation of *Escherichia coli* O157:H7 from retail fresh meats and poultry. Appl Environ Microbiol 1987;53:2394-6.
6. Sanderson MW, Gay JM, Hancock HH, Gay CC, Fox LK, Besser TE. Sensitivity of bacteriologic culture for detection of *Escherichia coli* O157:H7 in bovine feces. J Clin Microbiol 1995;33:2616-9.
7. Okrend AJG, Rose BE, Lattuada CP. Isolation of *Escherichia coli* O157:H7 using O157 specific antibody coated magnetic beads. J Food Prot 1992;55:214-7.
8. Olsvik O, Strockbine NA. PCR detection of heat-stable heat-labile, and Shiga-like toxin genes in *Escherichia coli*. In: Persing DH, Smith TF, Tenover FC, White TJ, editors. Diagnostic molecular microbiology. Washington: American Society for Microbiology; 1993.
9. Cebula TA, Payne WL, Feng P. Simultaneous identification of strains of *Escherichia coli* serotype O157:H7 and their Shiga-like toxin type by mismatch amplification mutation assay-multiplex PCR. J Clin Microbiol 1995;33:248-50.
10. Tauxe RV, Kruse H, Hedberg C, Potter M, Madden J, Wachsmuth K. Microbial hazards and emerging issues associated with produce. A preliminary report to the national advisory committee on microbiologic criteria for foods. J Food Prot 1997;60:1400-8.
11. Mahon BE, Ponka A, Hall WN, Komatsu K, Dietrich SE, Siitonen A, et al. An international outbreak of *Salmonella* infections caused by alfalfa sprouts grown from contaminated seeds. J Infect Dis 1997;175:876-82.
12. van Beneden CA, Keene WE, Strang RA, Werker DH, King AS, Mahon B, et al. Multinational outbreak of *Salmonella enterica* serotype Newport infections due to contaminated alfalfa sprouts. JAMA 1999;281:158-62.
13. Mitscherlich E, Marth EH. Microbial survival in the environment. New York: Springer-Verlag; 1984.
14. Itoh Y, Sugita-Konishi Y, Kasuga F, Iwaki M, Hara-Kudo Y, Saito N, et al. Enterohemorrhagic *Escherichia coli* O157:H7 present in radish sprouts. Appl Environ Microbiol 1998;64:1532-5.
15. Kurtzweil P. Questions keep sprouting about sprouts. Food and Drug Administration Consumer Magazine 1999;33:18-22.
16. U.S. Food and Drug Administration. Interim advisory on alfalfa sprouts. Rockville (MD): The Administration; 1998. T98-47.
17. California Department of Health Services. State Health Department interim advisory on raw alfalfa sprouts. Sacramento: California Department of Health Services Office of Public Affairs; 1998. p. 81-98.
18. Wang G, Doyle MP. Survival of enterohemorrhagic *Escherichia coli* O157:H7 in water. J Food Prot 1998;61:662-7.
19. Akashi S, Joh K, Tsuji A, Ito H, Hoshi H, Hayakawa T, et al. A severe outbreak of haemorrhagic colitis and haemolytic uremic syndrome associated with *Escherichia coli* O157:H7 in Japan. Eur J Pediatr 1994;153:650-5.
20. Keene WE, McAnulty JM, Hoesly FC, Williams L Jr, Hedberg K, Oxman GL, et al. A swimming-associated outbreak of hemorrhagic colitis caused by *Escherichia coli* O157:H7 and *Shigella sonnei*. N Engl J Med 1994;331:579-84.
21. Tauxe RV. *Salmonella*: a postmodern pathogen. J Food Prot 1991;54:563-8.
